# Blackcurrant Alters Physiological Responses and Femoral Artery Diameter during Sustained Isometric Contraction

**DOI:** 10.3390/nu9060556

**Published:** 2017-05-29

**Authors:** Matthew David Cook, Stephen David Myers, Mandy Lucinda Gault, Mark Elisabeth Theodorus Willems

**Affiliations:** 1 Department of Sport & Exercise Sciences, University of Chichester, College Lane, Chichester PO19 6PE, UK; matthew.cook@worc.ac.uk (M.D.C.); s.myers@chi.ac.uk (S.D.M.); m.gault@chi.ac.uk (M.L.G.); 2 Institute of Sport and Exercise Science, University of Worcester, Henwick Grove, Worcester WR2 6AJ, UK

**Keywords:** cardiovascular function, anthocyanins, blood flow, isometric contraction, New Zealand blackcurrant, electromyography, ultrasound, exercise

## Abstract

Blackcurrant is rich in anthocyanins that may affect exercise-induced physiological responses. We examined tissue oxygen saturation, muscle activity, cardiovascular responses and femoral artery diameter during a submaximal sustained isometric contraction. In a randomised, double-blind, crossover design, healthy men (*n* = 13, age: 25 ± 4 years, BMI: 25 ± 3 kg·m^−2^, mean ± SD) ingested New Zealand blackcurrant (NZBC) extract (600 mg∙day^−1^ CurraNZ™) or placebo (PL) for 7-days separated by 14-days washout. Participants produced isometric maximal voluntary contractions (iMVC) and a 120-s 30%iMVC of the *quadriceps* with electromyography (EMG), near-infrared spectroscopy, hemodynamic and ultrasound recordings. There was no effect of NZBC extract on iMVC (NZBC: 654 ± 73, PL: 650 ± 78 N). During the 30%iMVC with NZBC extract, total peripheral resistance, systolic, diastolic, and mean arterial pressure were lower with increased cardiac output and stroke volume. With NZBC extract, EMG root mean square of the *vastus medialis* and muscle oxygen saturation were lower with higher total haemoglobin. During the 30%iMVC, femoral artery diameter was increased with NZBC extract at 30 (6.9%), 60 (8.2%), 90 (7.7%) and 120 s (6.0%). Intake of NZBC extract for 7-days altered cardiovascular responses, muscle oxygen saturation, muscle activity and femoral artery diameter during a 120-s 30%iMVC of the *quadriceps*. The present study provides insight into the potential mechanisms for enhanced exercise performance with intake of blackcurrant.

## 1. Introduction

Blackcurrant contains a high and specific content of anthocyanins [1], considered to be the essential bioactive berry compounds. New Zealand blackcurrant (NZBC) altered cardiovascular function in rest by increased cardiac output [2,3], and improved cycling endurance [4] and repeated high-intensity running performance [5]. However, the mechanisms for the ergogenic effects of New Zealand blackcurrant are unknown. Matsumoto et al. [6] observed with blackcurrant intake an increase in forearm blood flow at rest following arterial occlusion, and a higher change in total haemoglobin in the trapezius muscle during a maximal voluntary contraction (MVC) after 30 min of typing, measured by infrared spectroscopy (NIRS). Such observations may be mediated by the blackcurrant anthocyanins (or metabolites) influencing vasodilation and relaxation [7], by increasing production of nitric oxide. Other in vivo studies also observed anthocyanins to vasodilate blood vessels by increasing flow-mediated dilation at rest [8,9]. However, the potential effects of NZBC on enlarging blood vessel diameter during exercise have not been examined.

It is important to note that Matsumoto et al. [6] reported the increase in peripheral blood flow of the forearm from a rate of increase in total haemoglobin after releasing an occlusion by a pressured cuff around the upper arm. However, NIRS only provides a proxy measure of blood flow as a change would assume muscle perfusion from an artery to be greater, i.e., as a result of vasodilation or increased flow rate.

Because muscle blood flow is of critical importance in oxygen delivery for muscle metabolism, an impediment of blood flow will precipitate fatigue. Blood flow into muscle is sensitive to the intensity and type of contraction [10,11,12]. Sustained isometric contractions present a challenge to muscle blood perfusion as the increased demand for flow is counteracted by the increased intramuscular pressure. For example, it has been observed that with muscle forces above 30% of MVC, blood flow becomes impaired as intramuscular pressure rises above that of systolic blood pressure in the elbow flexors, knee extensors and plantar flexors [11] and handgrip flexors [12]. McNeil et al. [10] also observed that there was no change in anterior tibial artery diameter during a 60-s isometric contraction at 30% of MVC, but it was significantly compressed at 60% and 100% of MVC. Therefore, sustained isometric exercise presents a challenge to blood flow and nutritional interventions that can alter responses to benefit blood flow are potentially of interest to athletes undertaking exercise where prolonged isometric force production is required.

Motor unit behaviour during sustained isometric contractions indicates a decline in firing rate, with the recruitment of additional motor units as the contraction duration continues [13]. It has been suggested that the decrease in motor unit firing rate is mediated by chemoreceptive small diameter afferent nerves (groups III, IV) [13,14]. Afferent III and IV nerves respond to by-products of muscle contraction [15,16]. Therefore, during a submaximal isometric contraction, an increase in vasodilation of an artery by NZBC supplying an exercising muscle would be expected to increase muscle perfusion and lead to a reduction in by-products acting upon these afferent nerves. This would then be expected to lower recruitment of muscle fibres to compensate for fatigue within other fibres and in turn, result in a reduced root mean square (RMS) measured during electromyography. The effect of blackcurrant upon motor unit behaviour during isometric contraction is unknown.

The aim of the present study was to examine the effect of a New Zealand blackcurrant extract on blood vessel diameter, cardiovascular responses, muscle activity, and muscle oxygen saturation during a sustained submaximal isometric contraction.

## 2. Materials and Methods

### 2.1. Participants

Thirteen men (age: 25 ± 4 years (range 21–35 years), height: 182 ± 6 cm, body mass: 82 ± 9 kg, body fat: 13 ± 3%, BMI: 25 ± 3 kg·m^−2^ (range 20.6–29.9 kg·m^−2^, six with BMI between 18.5 and 24.9, seven with BMI between 25 and 29.9) provided written informed consent to participate in the study. Participants were healthy, physically active, non-smokers, and without history of musculoskeletal injury. Participants were not involved in a structured training programme at the time of the study and were not taking dietary supplements and prescription and non-prescription drugs. The study was approved by the University of Chichester Research Ethics Committee (approval code: 1617_38) with protocols and procedures performed in accordance with the ethical principles outlined by the Declaration of Helsinki (World Medical Association, 2013).

### 2.2. Experimental Design

Participants visited the laboratory for 3 visits, at the same time of day (~8:00 am). During the first visit, height (Harpenden Wall Mounted Stadiometer, UK), body mass (Kern ITB, Kern, Germany) and body fat were measured (Tanita BC418 Segmental Body Composition analyzer, Tanita, IL, USA). A testing bench was adjusted to fit participants so that hip and knee angle was 90°. The femoral artery was then identified by ultrasound (MicroMaxx Doppler ultrasound, Sonosite, Inc; Bothwell, WA, USA) for measurement of resting diameter (method adapted from Shoemaker et al. [17]). Electromyography electrodes were placed upon the skin to record signals from the *vastus medialis*, *rectus femoris* and *biceps femoris muscles*. This was followed by participants completing three maximal voluntary contractions of the knee extensors with EMG recording (Delsys Bagnoli-8 system, Delsys Inc., Boston, MA, USA). A line was placed upon the computer screen to represent 30%iMVC (i.e., isometric maximal voluntary contraction) (calculated from the highest force during the 3 iMVCs), which participants produced for 120 s. During the sustained isometric contraction, whole body cardiovascular measurements were recorded using a beat-to-beat blood pressure monitoring system (Portapres^®^ Model 2, Finapres Medical Systems BV, Amsterdam, The Netherlands), muscle oxygen saturation of the *rectus femoris muscle* measured using NIRS (Moxy Monitor, Hutchinson, MN, USA), EMG recorded and diameter of the femoral artery measured by ultrasound. The first visit allowed participants to become familiarised with all testing procedures before the experimental conditions in visits 2 and 3.

For 7-days prior to visits 2 and 3, participants consumed 2 × 300 mg capsules (total 210 mg of anthocyanins) of NZBC extract (CurraNZ™, Health Currancy Ltd, Surrey, UK) or identical looking placebos (2 × 300 mg microcrystalline cellulose M102) every morning with breakfast. Each capsule of 300 mg contains 105 mg of anthocyanins, i.e., 35%–50% delphinidin-3-rutinoside, 5%–20% delphinidin-3-glucoside, 30%–45% cyanidin-3-rutinoside, and 3%–10% cyanidin-3-glucoside with remaining content mainly natural plant sugars. The dose used was established from a previous study in which a dose-response relationship in cardiovascular responses was observed in trained cyclists [2]. Optimal dosing strategy for New Zealand blackcurrant extract is not known. However, previous studies on effects of fruit juices dosed also for multiple days before exercise testing (e.g., 4 days tart cherry juice [18], 6 days tart cherry juice [19], and 8 days montmorency cherry juice [20]). On the final day of supplementation in the present study, participants reported to the laboratory, two hours post-prandial of a standard breakfast (i.e., one slice of buttered bread or toast ~840 kJ, ~30 g carbohydrate, ~6 g protein and ~7 g fat) and the capsules required for that condition. The two experimental conditions (NZBC extract and placebo) were performed in a randomised double-blind, cross-over design with a 14-day washout period. Seven participants received NZBC extract as the first condition.

### 2.3. Isometric Maximum Voluntary Contraction

A metal cuff with soft strap was attached to the ankle of the participant proximal to the fibular notch and medial malleolus and attached via steel chain to an s-beam load cell (RS 250 kg, Teda Hutleigh Cardiff, UK). Participants then completed three warm up isometric contractions (~50% MVC held for 5 s) prior to performing three isometric MVCs with standardised instructions [21]. Each iMVC lasted approximately 3–4 s with the highest mean force produced for 0.5 s during the three contractions taken as iMVC force. EMG measurements were recorded during each iMVC. Between MVCs, participants rested for 2 min. A screen displaying force was placed in front of participants and recorded on computer at 1000 Hz using Chart 4 V4. 1.2 (AD Instruments, Oxford, UK). All testing was performed with the dominant leg.

### 2.4. Ultrasound of Femoral Artery

The femoral artery was insonated by ultrasound (MicroMaxx portable ultrasound, Sonosite, Bothell, WA, USA) with an 8-Mhz linear array transducer in B-mode and an angle of approach at 90°. Participants were scanned while sitting on the isometric chair with the hip angle at 90°, and with the probe in the transverse plane, approximately 7 cm below the inguinal ligament to avoid the femoral artery bifurcation. Arterial diameter measurements were made from the average of 3 frozen images during diastole [17] at rest and 30, 60, 90 and 120 s during the sustained isometric contraction.

### 2.5. Near Infrared Spectroscopy

The NIRS was taped centrally on the *rectus femoris* and recorded at a sampling frequency of 2 Hz and at wavelengths of 630 nm and 850 nm. Muscle oxygen saturation (SmO_2_%) and haemoglobin concentration (THb) was measured at rest and 15, 30, 45, 60, 75, 90, 105 and 120 s in the sustained isometric contraction, with six measurements averaged and centred around each time point.

### 2.6. Electromyography

Electromyography signals (bandwidth = 20 to 240 Hz; common mode rejection ratio = 92 dB; input impedance ≥ 1015 Ω) of the *vastus medialis*, *rectus femoris* and *biceps femoris* were taken during the iMVC and sustained isometric contraction. To ensure accurate electrode positioning in all trials, the recommendations for placement followed guidelines by SENIAM (http://www.seniam.org/). The skin was prepared by shaving, cleansing and abrading to minimize skin-to-electrode impedance. The electrode was placed transverse to the muscle fibre pennation angle and attached the skin with tape. A reference electrode was placed approximately 4 cm proximal to the patella apex of the leg measured.

EMG data were collected and processed using Delsys EMGworks^®^ Acquisition and Analysis software (Delsys INC, Boston, MA, USA). Raw EMG signals were amplified at 1000 Hz, and then filtered using a 2nd order Butterworth bandpass filter (low 10 Hz, high 350 Hz). The Root Mean Square (RMS) of the filtered signal (mV) was calculated using a moving window (window length 0.05 s, window overlap 0.025 s). The RMS data are expressed as normalized values from the highest RMS value sustained for 0.5 s during the iMVC. During the 120-s contraction, the calculated RMS values were averaged around the centred time point (e.g., 14.5–15.5 s) for the 15, 30, 45, 60, 75, 90, 105 and 120 s during the sustained isometric contraction. From the filtered signal, the median frequency (MDF) was calculated with a moving window (window length: 1 s, window overlap: 0.5 s). The mean slope by linear regression analysis of the MDF was calculated for all values of the sustained isometric contraction.

### 2.7. Cardiovascular Measurements

Cardiovascular responses were recorded using a beat-to-beat blood pressure monitoring system during 10 min of rest in a sitting position using the arterial volume clamp method [22]. The Portapres^®^ is a beat-to-beat finger blood pressure analyser that allows the non-invasive continuous measurement of haemodynamic parameters. The finger cuff was positioned around the same finger of the left hand. Cardiovascular recordings in rest were averaged over 10 consecutive beats, with the lowest systolic blood pressure and associated measures recorded. This approach avoids selection of signal content. During the sustained isometric contraction, the participants were instructed to keep the left hand stationary in their lap. Cardiovascular recordings during the sustained isometric contraction were averaged over 6 consecutive beats centred around each time point of the 120 s contraction (i.e., 15, 30, 45, 60, 90, 105 and 120 s). The following parameters were derived: stroke volume, cardiac output, systolic blood pressure, diastolic blood pressure, mean arterial blood pressure, ejection time, and total peripheral resistance (Beatscope 1.1a., Finapres Medical Systems BV, Amsterdam, The Netherlands).

### 2.8. Physical Activity and Dietary Standardisation

Participants were instructed to keep their weekly exercise schedule as consistent as possible. Before all visits to the laboratory, participants were instructed not to exercise and consume alcohol 24 h before, not to consume caffeine 4 h before, and not take other dietary supplements that add further nutritional value to the normal diet.

Participants recorded their dietary intake and exercise on a written diary 48 h prior to the first experimental condition (i.e., visit 2) and were instructed for the subsequent experimental visit (i.e., visit 3) to replicate intake using the diary as a guide, while recording on a new diary their dietary intake for that visit. Participants confirmed adherence to the study criteria at the start of every visit. Food diaries were analysed using Nutritics (Nutritics LTD, Dublin, Ireland) for carbohydrate, fat and protein intake and total energy intake (kJ). There were no differences (*p* > 0.05) in absolute values or relative values (i.e., per kilogram of body mass) for carbohydrate, fat, protein, or total energy intake for 48 h prior to each experimental visit (Table 1). Analysis of the food diaries identified that all participants reported 100% adherence to the dietary instructions 48 h prior to each visit.

### 2.9. Statistical Analysis

Statistical analyses were completed using SPSS 20.0 (SPSS, Chicago, IL, USA). Data normality assumptions were assessed using Kolmogorov-Smirnov test. Paired samples t-tests used were to compare maximal force during the iMVCs, mean of the sustained isometric force during the 30%iMVC, resting cardiovascular function, resting muscle oxygen saturation, femoral artery diameter and the 48-h dietary intake between the NZBC and placebo conditions. Differences between cardiovascular function and EMG during the sustained isometric contraction were analysed using a condition (control vs. NZBC) by time-point (15, 30, 45, 60, 75, 90, 105, and 120 s) repeated measures analysis of variance (ANOVA) with LSD *post hoc* comparisons. Differences between the femoral artery diameter during the sustained contraction were analysed using a condition by time-point (30, 60, 90 and 120 s) repeated measures ANOVA with LSD *post hoc* comparisons. Mauchley’s Test of Sphericity was conducted to test for homogeneity of data and where violations were present; Greenhouse-Geiser adjustments were made. To determine the effect size of responses, Cohen’s *d* were calculated [23]. Cohen [23] described an effect size of <0.2 as a trivial, 0.2–0.39 as a small, 0.4–0.69 as a moderate and ≥0.7 as a large magnitude of change. From the 15% change in cardiac output following 600 mg∙day^−1^ of NZBC extract in Cook et al. [2], an a-priori power analysis indicated a sample size of 13 would allow a detection of a 15% increase in cardiac output with a high statistical power (1−β = 0.95: 0.05 = α level). All data are reported as mean ± SD and significance was accepted at *p* < 0.05.

## 3. Results

### 3.1. Isometric Maximal Voluntary Contraction and Sustained Isometric Force

There was no effect of NZBC on maximal isometric voluntary contraction force (NZBC: 654 ± 73, PL: 650 ± 78 N, *p* = 0.732) or average force during the sustained isometric contraction for 120 s (NZBC: 182 ± 24, PL: 182 ± 23 N, *p* = 0.934).

### 3.2. Cardiovascular Function in Rest

At rest, before the sustained isometric contraction, there were no differences in systolic blood pressure (NZBC: 126 ± 11, PL: 126 ± 11 mmHg, *p* = 0.901), diastolic blood pressure (NZBC: 75 ± 10, PL: 72 ± 9 mmHg, *p* = 0.319), mean arterial pressure (NZBC: 91 ± 10, PL: 89 ± 10 mmHg, *p* = 0.553), heart rate (NZBC: 75 ± 8, PL: 71 ± 10 beats∙min^−1^, *p* = 0.204) and ejection time (NZBC: 0.27 ± 0.02, PL: 0.27 ± 0.01 s, *p* = 0.330). Cardiac output (NZBC: 6.6 ± 1.6, PL: 5.5 ± 1.4 L∙min^−1^, *p* = 0.001, *d* = 0.73) and stroke volume (NZBC: 90 ± 17, PL: 80 ± 18 mL, *p* = 0.015, *d* = 0.57) were increased by 16% and 11%, respectively. There was a 25% lower total peripheral resistance (NZBC: 14.3 ± 4.1, PL 17.3 ± 4.2 mmHg∙L^−1^∙min^−1^, *p* = 0.003, *d* = 0.72). The changes in resting cardiovascular function were observed in 12 participants.

### 3.3. Cardiovascular Responses during the Sustained Isometric Contraction

**Figure 1:**
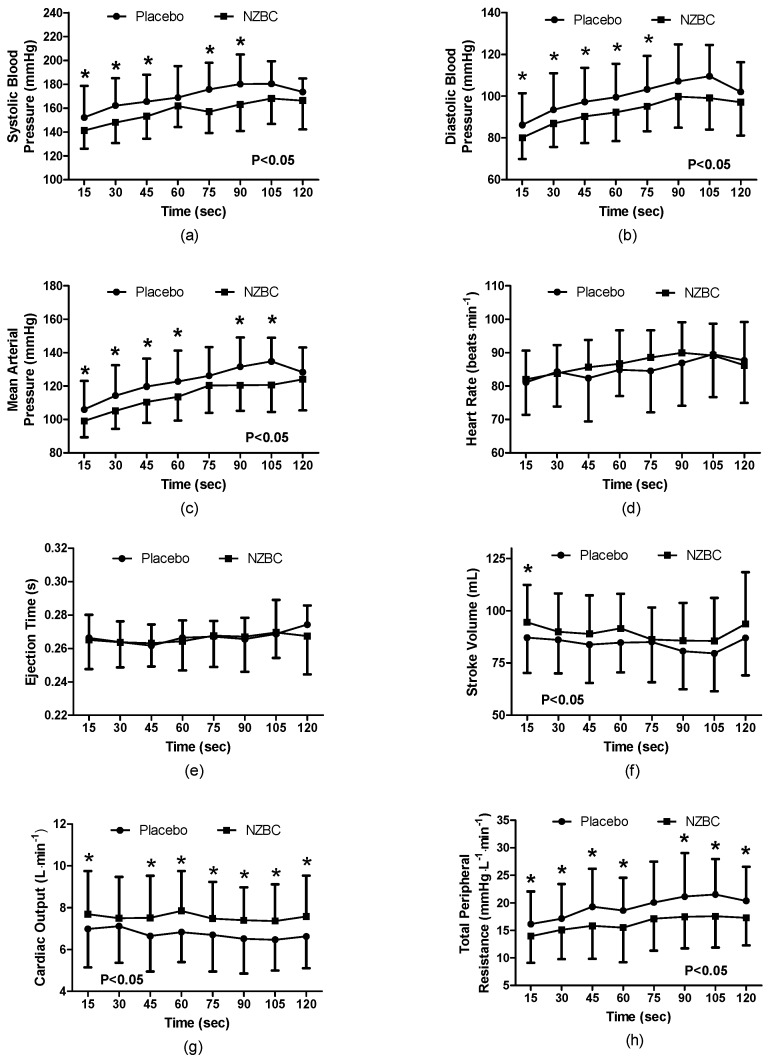
(**a**) Systolic Blood Pressure; (**b**) Diastolic Blood Pressure; (**c**) Mean Arterial Pressure; (**d**) Heart Rate; (**e**) Ejection Time; (**f**) Stroke Volume; (**g**) Cardiac Output; (**h**) Total Peripheral Resistance during a 120-s 30%iMVC of the *quadriceps muscle* for placebo and after 7-days intake of NZBC (New Zealand blackcurrant) extract capsules. Data are mean ± SD. * difference between placebo and NZBC extract (*p* < 0.05).

During the 120 s isometric contraction, systolic blood pressure was different between the conditions (F_(1,96)_ = 41.41, *p* < 0.001) with a time effect (F_(7,96)_ = 3.28, *p* = 0.004) and no interaction effect (F_(7,96)_ = 0.58, *p* = 0.778). With NZBC, systolic pressure was lower at 15 (7%, *p* = 0.036, *d*=0.48), 30 (10%, *p* = 0.020, *d* = 0.68), 45 (9%, *p* = 0.023, *d* = 0.60), 75 (12%, *p* = 0.003, *d* = 0.92) and 90 s (11%, *p* = 0.008, *d* = 0.73) (Figure 1a). At 105 s, there was a trend for systolic pressure to be lower with NZBC (*p* = 0.083). Diastolic pressure was different between the conditions (F_(1,96)_ = 35.27, *p* < 0.001) with a time effect (F_(7,96)_ = 3.65, *p* = 0.002) and no interaction effect (F_(7,96)_ = 0.21, *p* = 0.982). With NZBC, diastolic pressure was lower at 15 (7%, *p* = 0.020, *d* = 0.46), 30 (7%, *p* = 0.044, *d* = 0.30), 45 (8%, *p* = 0.022, *d* = 0.47), 60 (8%, *p* = 0.011, *d* = 0.47) and 75 s (9%, *p* = 0.008, *d* = 0.56). There were trends for diastolic pressure to be lower with NZBC at 90 (*p* = 0.060) and 105 s (*p* = 0.064) (Figure 1b). Mean arterial pressure was different between the conditions (F(_1,96)_ = 39.55, *p* < 0.001) with a time effect (F_(7,96)_ = 5.37, *p* < 0.001) and no interaction effect (F_(6,96)_ = 0.62, *p* = 0.735). NZBC decreased mean arterial pressure at 15 (7%, *p* = 0.022, *d* = 0.44), 30 (9%, *p* = 0.020, *d* = 0.58), 45 (9%, *p* = 0.009, *d* = 0.59), 60 (8%, *p* = 0.008, *d* = 0.52), 90 (10%, *p* = 0.008, *d* = 0.76) and 105 s (13%, *p* = 0.018, *d* = 0.89) (Figure 1c).

Heart rate was not different between the conditions (F_(1,96)_ = 1.61, *p* = 0.207), with no time (F_(7,96)_ = 1.02, *p* = 0.420) or interaction effect (F_(7,96)_ = 0.45, *p* = 0.868) (Figure 1d). Similarly, ejection time was not different between the conditions (F_(1,96)_ = 0.16, *p* = 0.688), with no time (F_(7,96)_ = 0.61, *p* = 0.744), or interaction effect (F_(7,96)_ = 0.27, *p* = 0.963) (Figure 1e).

Stroke volume was different between the conditions (F_(1,96)_ = 9.81, *p* = 0.002), with no time (F_(7,96)_ = 0.47, *p* = 0.865) or interaction effect (F_(7,96)_ = 0.17, *p* = 0.990). NZBC increased stroke volume at 15 s (7%, *p* = 0.048, *d* = 0.37) (Figure 1f). Cardiac output was different between the conditions (F_(1,96)_ = 38.81, *p* < 0.001), with no time (F_(7,96)_ = 0.14, *p* = 0.999) or interaction effect (F_(7,96)_ = 0.29, *p* = 0.955). NZBC increased cardiac output at 15 (8%, *p* = 0.048, *d* = 0.37), 45 (9%, *p* = 0.056, *d* = 0.60), 60 (10%, *p* = 0.033, *d* = 0.60), 75 (10%, *p* = 0.029, *d* = 0.45), 90 (12%, *p* = 0.016, *d* = 0.58), 105 (10%, *p* = 0.034, *d* = 0.55) and 120 s (11%, *p* = 0.013, *d* = 0.54) (Figure 1g). Total peripheral resistance was different between the conditions (F_(1,96)_ = 42.60, *p* = 0.001), with no time (F_(7,96)_ = 1.05, *p* = 0.404) or interaction effect (F_(7,96)_ = 0.25, *p* = 0.972). NZBC decreased total peripheral resistance at 15 (17%, *p* = 0.012, *d* = 0.41), 30 (16%, *p* = 0.085, *d* = 0.35), 45 (25%, *p* = 0.040, *d* = 0.55), 60 (25%, *p* = 0.031, *d* = 0.50), 90 (21%, *p* = 0.019, *d* = 0.38), 105 (29%, *p* = 0.042, *d* = 0.65) and 120 s (19%, *p* = 0.024, *d* = 0.54) (Figure 1h).

### 3.4. Electromyography

**Figure 2:**
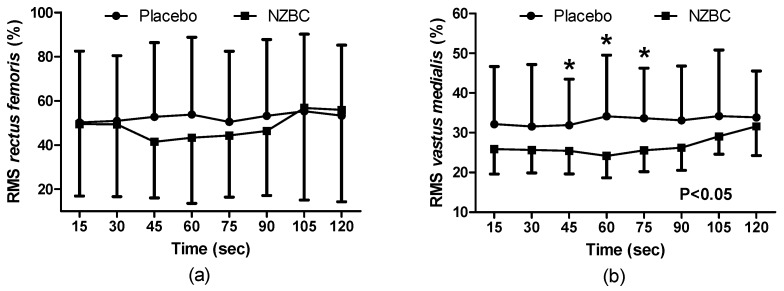
Normalised root mean square (RMS) of the *rectus femoris muscle* (**a**) and the *vastus medialis muscle* (**b**) during a 120 s 30%iMVC for placebo and after 7-days intake of NZBC (New Zealand blackcurrant) extract capsules. Data are mean ± SD. * difference between placebo and NZBC extract.

During the sustained isometric contraction there was no condition (F_(1,96)_ = 0.59, *p* = 0.444), time (F_(7,96)_ = 0.39, *p* = 0.905) or interaction effect (F_(7,96)_ = 0.12, *p* = 0.996) on normalised EMG of muscle activation for the *rectus femoris* (Figure 2a). The normalised EMG for the *vastus medialis* showed a condition effect (F_(1,96)_ = 22.04), *p* < 0.001), with no time (F_(7,96)_ = 0.43, *p* = 0.881) or interaction effect (F_(7,96)_ = 0.34, *p* = 0.935), with NZBC decreasing normalised RMS at 45 (16%, *p* = 0.050, *d* = 0.68), 60 (12%, *p* = 0.034, *d* = 0.75) and 75 s (11%, *p* = 0.015, *d* = 0.75) (Figure 2b). For the *biceps femoris*, there was no condition (F_(1,96)_ = 0.72, *p* = 0.399), time (F_(7,96)_ = 0.08, *p* = 0.999) or interaction effect (F_(7,96)_ = 0.04, *p* = 0.999).

There was no difference in the slope of the MDF during the sustained contraction for the *rectus femoris* (NZBC: −0.030 ± 0.050, PL: −0.036 ± 0.073, *p* = 0.788), *vastus medialis* (NZBC: 0.010 ± 0.023, PL: 0.008 ± 0.053, *p* = 0.879) or *biceps femoris* (NZBC: 0.012 ± 0.054, PL: 0.011 ± 0.049, *p* = 0.955).

### 3.5. Near Infrared Spectroscopy

At rest, there was a strong trend for SmO_2_% to be lower following NZBC (NZBC: 64 ± 12, PL: 70 ± 11%, *p* = 0.067) with no difference in THb concentration (NZBC: 12.4 ± 0.4, PL: 12.2 ± 0.3 g∙dL^−1^, *p* = 0.145).

**Figure 3:**
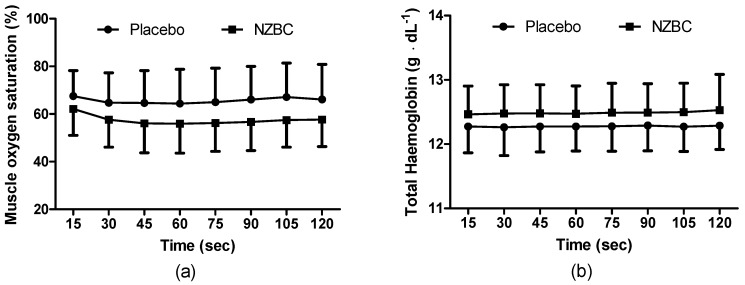
(**a**) Muscle oxygen saturation; (**b**) total haemoglobin concentration during a 120-s 30%iMVC of the *quadriceps muscle* for placebo and after 7-days intake of NZBC (New Zealand blackcurrant) extract capsules. Data are mean ± SD. * difference between placebo and NZBC extract.

During the sustained isometric contraction, SmO_2_% was different between the conditions (F_(1,96)_ = 30.12, *p* < 0.001) with no time (F_(7,96)_ = 0.29, *p* = 0.958) or interaction effect (F_(7,96)_ = 0.10, *p* = 0.997) (Figure 3a). THb was different between the conditions (F_(1,96)_ = 15.55, *p* < 0.001) with no time (F_(7,96)_, *p* = 1.000) or interaction effect (F_(7,96)_ = 0.01, *p* = 1.000) (Figure 3b). There were no time points different for either measure.

### 3.6. Femoral Artery Diameter

There was no difference in femoral artery diameter at rest (NZBC: 0.75 ± 0.07, PL: 0.75 ± 0.09 cm, *p* = 0.902) or 30 s following the sustained isometric contraction (NZBC: 0.75 ± 0.12, PL: 0.76 ± 0.12 cm, *p* = 0.687) for either experimental condition.

**Figure 4:**
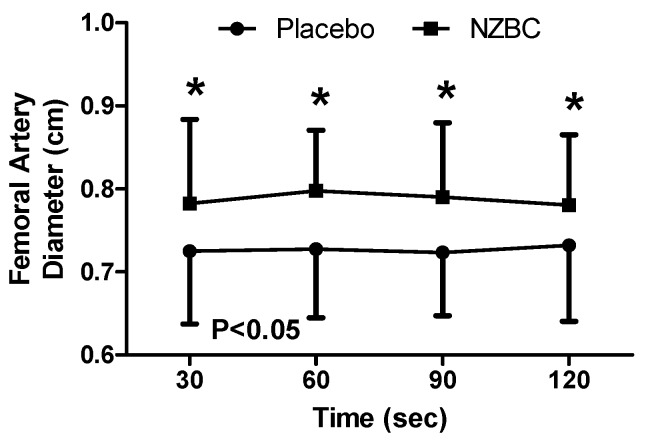
Femoral artery diameter during a 120-s 30%iMVC of the *quadriceps muscle* for placebo and after 7-days intake of NZBC (New Zealand blackcurrant) extract capsules. Data are mean ± SD. * difference between placebo and NZBC extract.

During the sustained isometric contraction, there was a difference between the conditions (F_(1,48)_ = 35.56, *p* < 0.001), with no difference observed across time (F_(3,48)_ = 0.03, *p* = 0.993) or an interaction effect (F_(3,48)_ = 0.23, *p* = 0.874). *Post hoc* testing identified femoral artery diameter to be greater with NZBC (Figure 4) at 30 (*p* = 0.009, *d* = 0.52), 60 (*p* = 0.003, *d* = 0.80), 90 (*p* = 0.021, *d* = 0.82) and 120 s (*p* = 0.022, *d* = 0.58) with increases of 6.9% (12 of 13 participants increased), 8.2% (12 of 13 participants increased), 7.7% (11 of 13 participants increased) and 6.0% (11 of 13 participants increased).

## 4. Discussion

The principal finding from the present study was that femoral artery diameter was increased with intake of NZBC extract during a submaximal (i.e., 30% iMVC) 120-s sustained isometric contraction of the *quadriceps muscles*. The enlarged diameter of the femoral artery was accompanied by alterations in cardiovascular responses with a decrease in systolic and diastolic blood pressure, mean arterial blood pressure and total peripheral resistance with a concomitant increase in cardiac output and stroke volume. The normalised RMS for the *vastus medialis* and muscle oxygen saturation was also decreased while total haemoglobin concentration in the muscle was increased by the NZBC extract.

Regardless of the experimental condition within the present study (i.e., NZBC extract or placebo), the physiological responses during the sustained isometric contraction are similar to those of McNeil et al. [10]. For example, heart rate was unchanged while mean arterial blood pressure increased over time during an isometric contraction at 30% of MVC [10]. The cardiovascular function responses in the study by McNeil et al. [10] also occurred without an alteration in artery diameter (i.e., no observed time effect), or a change in normalised RMS during the contraction. An interesting observation from the present study is that NZBC increased femoral artery in comparison to a placebo at every time point during the contraction, however before and after the contraction, the diameter was not different.

The effect of the anthocyanins from the NZBC on vascular function and arterial diameter during the isometric contraction are thought to occur from them modulating an increase in concentration of nitric oxide [24,25]. However, as anthocyanins are reported to have low bioavailability [26], the bioactivity is likely mediated by the metabolites, that exist within the circulation at much higher concentrations [27,28]. An intake of anthocyanins with a similar dose to the present study increased flow-mediated dilation (FMD) 1 h following intake [8]. This occurred alongside a peak in the anthocyanin metabolites, ferulic acid, vanillic acid, isoferulic acid, 2-hydroxybenzoic acid and caffeic acid and a reduction in neutrophil NADPH oxidase. What is more, Edwards et al. [29] reported that the anthocyanin cyanidin-3-glucoside could increase eNOS expression, whereas the metabolites protocatechuic acid and vanillic acid did not. However, these metabolites elicited a reduction in superoxide production, which could decrease scavenging of nitric oxide. Therefore, while the anthocyanin can increase eNOS expression, metabolism of the anthocyanin into its metabolites loses this effect. However, the metabolites can maintain vascular homeostasis by increasing nitric oxide bioactivity through mechanisms involving NADPH inhibition or inducing cytoprotective enzyme haem oxygenase-1 (HO-1), an enzyme that catalyzes the degradation [29]. Similarly, Keane et al. [30] observed in pre-hypertensive males following 60 mL of Montmorency cherry (~62 mg anthocyanin) a peak reduction in systolic blood pressure of 7 ± 2 mmHg 2 h following intake, which occurred at the same time point as peak increases in the metabolites protocatechuic acid and vanillic acid within the plasma. Interestingly within this *in vivo* study, Keane et al. [30] observed no effect on plasma nitrate and nitrite (a proxy measure of nitric oxide production), further suggesting the metabolites are responsible for the vascular response. Indeed, this was also shown by Keane et al. [30], whereby, human vascular smooth muscles migrated further in vitro in response to a combination of the metabolites protocatechuic and vanillic acid, rather than when incubated in isolation. However, it should be noted that Czank et al. [27] observed 24 metabolites from the anthocyanin cyanidin-3-glucoside in human serum, which may suggest that many metabolites have to be examined for cardiovascular bioactivity rather than a few which have currently been studied.

It is considered unlikely within the present study that the intramuscular pressure from the isometric contraction was large enough to reduce blood flow into the muscle. Isometric contractions above 30% of MVC have been observed to reduce artery diameter [10], however no time effect for the artery diameter indicates the diameter at this contraction force and duration is consistent. Shoemaker et al. [31] reported that the day-to-day variation of an artery diameter during exercise ranged from 2.9 ± 0.4% to 3.96 ± 0.5%. The increases of femoral artery diameter in the present study were 6.0–8.2% and above what would be expected within daily variation and therefore attributed to the intake of NZBC extract. The effect of intake of NZBC extract upon femoral artery diameter may also explain how the cardiovascular function was altered. It is postulated that the effect of intake of NZBC extract on vasodilation was not exclusive for the femoral artery; therefore, this would explain the lower total peripheral resistance, systolic and diastolic and mean arterial blood pressure. It is also noteworthy that some participants responded, while others did not. It is therefore possible that, similar to the findings of George et al. [32], NZBC anthocyanins influence vascular responses differently based upon genotype. George et al. [32] observed that vasodilation in the forearm following acute consumption of a high flavonoid fruit and vegetable puree drink was different for expressions of the eNOS gene Glu298Asp, whereby there was higher endothelium-dependent vasodilation in response to acetylcholine 180 min after intake in GG individuals compared to GT. Therefore, different expressions of this gene may explain the variation in responses in the present study. What is more, George et al. [32] measured vasodilation by laser Doppler imaging with iontophoresis on the forearm, therefore measurements made in a peripheral vessel are not representative of generalised endothelial function. Lastly, George et al. [32] measured acute responses and it is unknown if repeated supplementation over 7-days, as in the present study, would modulate these responses and is, therefore worth future study.

The present study provides novel observations on the effects of blackcurrant anthocyanins on NIRS parameters during submaximal isometric exercise. SmO_2_% by NIRS reflects the dynamic between O_2_ supply and O_2_ consumption [33]. Therefore, presuming a greater oxygen supply from increased femoral artery diameter and cardiac output in the present study, a consistently lower SmO_2_ (%) following intake of NZBC extract could indicate greater extraction and utilisation of oxygen. This would then potentially explain the reduced RMS during the sustained contraction as oxygen utilisation is greater and results in reduced fatigue [34]. Another indirect measure of an increase in blood flow by NZBC was an increase in THb from the NIRS. This measure is made from a combination of haemoglobin and myoglobin. Assuming that blood haemoglobin concentration and relative contribution of myoglobin remains constant between the 7-day visits, an increase in absolute THb in the present study would indicate an increased amount of blood in the muscle. Although at rest, Bailey et al. [35] observed an increased THb following intake of beetroot with an elevated oxygenated haemoglobin response in the first 120 s and a reduced amplitude of the deoxygenated response by 13% during 360 s of moderate intensity cycling (80% of the gas exchange threshold). Unfortunately, the NIRS device used within the present study does not report deoxygenated values (combination of haemoglobin and myoglobin), therefore this comparison is not possible. However, within the study by Bailey et al. [35], the pulmonary oxygen uptake was reduced by 19%, indicating that less O_2_ extraction was required as a consequence of reduced aerobic energy turnover or muscle energy utilisation. The association between decreased saturation, potentially indicating greater extraction, and increased THb with intake of NZBC extract may suggest more complex mechanisms. For example an increased extraction could be postulated to be a result of increased VO_2_. From the Fick equation [36], an increase in O_2_ delivery (i.e., from an increase in cardiac output) would be expected to enable an increased uptake of O_2_. However, Willems et al. [2] and Cook et al. [4] did not observe any alteration in oxygen uptake during exercise following NZBC, therefore this warrants further investigation. In addition, the present study did not measure the pulmonary oxygen uptake response to the isometric contraction; therefore, this mechanism is speculative. In addition, the NIRS technique is not without limitations. Firstly, skin and adipose tissue thickness affect sensitivity due to contamination of the signal [37]. Consequently, individual differences could not be controlled for in the present study. Another issue is that the small volume of tissue measured does not represent the whole muscle. For example, activation, metabolism and most importantly perfusion are heterogeneously distributed within and between exercising muscles [38]. This may explain why the normalised EMG was not different for the *rectus femoris*, but it was different for the *vastus medialis*.

It is postulated that the increased femoral artery diameter and increase in total haemoglobin by NIRS represents an increase in blood flow. During a sustained isometric contraction, the metabolic condition within the muscle is a function of local blood flow [39]. An interesting observation in the present study was that for the *vastus medialis*, the intake of NZBC extract decreased amplitude as measured by normalised RMS within the EMG. The RMS provides an indicator of motor unit activity; therefore a reduction would indicate less recruitment of progressively larger, fatigued motor units compensating for the fatigue of currently active motor units [40]. A change in MDF values is considered a useful physiological indicator of muscle fatigue [41], therefore no change in the slopes from the linear regression analysis suggests that intake of NZBC extract had no effect on fatigue in the 120 s 30%iMVC. Furthermore, the slopes were very shallow (i.e., for the *vastus medialis* ~0.010), indicating that the sustained isometric contraction at this intensity and duration has a low fatiguing effect. A change to the slope to become steeper would be expected to occur from a decrease in membrane conduction velocity due to metabolic changes and ion shifts within the muscle [42]. However, this would only be expected for contractions in which blood flow was occluded or low-intensity isometric contractions to exhaustion.

### Limitations of the Study

Firstly, the present study took measures during a submaximal isometric contraction, consequently, we cannot be certain that the observations would occur during rhythmical exercise whereby the muscles contraction-relaxation phases are shorter and of different intensity. Secondly, the bioavailability of anthocyanins is low, however metabolites have been observed within the plasma 48 h following intake [27]. Therefore, bioaccumulation of metabolites may have occurred and caused the effects in the present study. However, as the last intake was taken 2-h before the measurements, it cannot be excluded that an acute effect from that intake caused the responses in the present study. While dietary intake for the 48 h before the experimental visits was replicated using food diaries, there was no restriction on fruit and vegetable intake. As polyphenol metabolites are known to act synergistically, habitual fruit and vegetable intake may have contributed to the changes observed. In addition, implementation of a wash-out diet (i.e., low phenolic diet for 48 h before each experimental visit) would indicate that the NZBC intake was the likely definitive factor causing the increase in femoral artery diameter, however such approach lowers ecological validity for those in an exercise performance setting. Future studies may also wish to measure antioxidant status of participants and quantify the polyphenol and anthocyanin intake of participants. Thirdly, we do not know the ultrasonographic lower limb atherosclerosis score (i.e., ULLA score) [43], but the ULLA score is likely zero as our participants were young adult, non-smoking and physically active men. It would be of interest to examine the effectiveness of New Zealand blackcurrant intake on vasodilation in peripheral arterial disease patients.

## 5. Conclusions

Seven days intake of 600 mg∙day^−1^ of New Zealand blackcurrant extract containing 210 mg∙day^−1^ anthocyanins, with the final intake 2 to 3 h before testing, increased vasodilation during sustained submaximal isometric exercise in young adult healthy men. This occurs alongside alterations in whole body cardiovascular responses, a decrease in muscle oxygen saturation and amplitude of electromyography signal, and an increase in total haemoglobin. Taken together, an increase in vasodilation and cardiac output would indicate an increase in peripheral blood flow, thereby providing support for a potential mechanism of improved exercise performance as observed in previous studies.

## Figures and Tables

**Table 1:** Absolute and relative to body mass dietary intake 48 h before each visit for placebo and NZBC extract condition.

Dietary Variable	Placebo	NZBC Extract
Carbohydrate (g)	499 ± 81	491 ± 73
(g∙kg body mass^−1^)	6.8 ± 1.4	6.7 ± 1.4
Fat (g)	230 ± 61	225 ± 69
(g∙kg body mass^−1^)	3.7 ± 1.1	3.9 ± 0.6
Protein (g)	219 ± 47	231 ± 46
(g·kg body mass^-1^)	2.4 ± 1.1	2.9 ± 0.9
Total energy intake (kJ)	20,764 ± 2835	20,799 ± 2981
(kJ·body mass^−1^)	284 ± 68	283 ± 61

NZBC, New Zealand blackcurrant; values are means ± SD for 13 participants.
